# TOM500: A Multi-Organ Annotated Orbital MRI Dataset for Thyroid Eye Disease

**DOI:** 10.1038/s41597-025-04427-9

**Published:** 2025-01-13

**Authors:** Haiyang Zhang, Hoi Chi Chan, Jiashuo Xu, Mengda Jiang, Xiaofeng Tao, Huifang Zhou, Xuefei Song, Xianqun Fan

**Affiliations:** 1 Department of Ophthalmology, Shanghai Ninth People’s Hospital, Shanghai Jiao Tong University School of Medicine, Shanghai, China; 2 Shanghai Key Laboratory of Orbital Diseases and Ocular Oncology, Shanghai, China; 3 Center for Basic Medical Research and Innovation in Visual System Diseases, Ministry of Education, Shanghai, China; 4 Department of Radiology, Shanghai Ninth People’s Hospital, Shanghai Jiao Tong University School of Medicine, Shanghai, China

**Keywords:** Ocular motility disorders, Thyroid diseases, Scientific data, Eye abnormalities

## Abstract

This study presents TOM500, a comprehensive multi-organ annotated orbital magnetic resonance imaging (MRI) dataset. It includes clinical data, T2-weighted MRI scans, and corresponding segmentations from 500 patients with thyroid eye disease (TED) during their initial visit. TED is a common autoimmune disorder with distinct orbital MRI features. Segmentations of nine orbital structures, including the optic nerve, orbital fat, lacrimal gland, eyeball, and five extraocular muscles (superior rectus and levator palpebrae superioris complex, inferior rectus, medial rectus, lateral rectus, and superior oblique), were generated by three junior annotators and reviewed by an expert radiologist. The consistency of the segmentations was evaluated using the intraclass correlation coefficient. Clinical data, including sex, age, disease duration, and smoking status, are also provided for disease diagnosis and classification. TOM500, the largest publicly available orbital MRI dataset with expert annotations, is designed to facilitate the development of advanced computational tools for TED diagnosis, classification, and observation.

## Background & Summary

Orbital magnetic resonance imaging (MRI) is an indispensable tool in the diagnosis and management of orbital diseases, particularly in the context of inflammatory conditions. Inflammatory orbital diseases, such as thyroid eye disease (TED), idiopathic orbital inflammation (IOI), and orbital cellulitis, present with distinct MRI characteristics^1,2^. In TED, MRI commonly shows enlarged extraocular muscles (EOMs) with increased T2-weighted signal intensity, indicative of edema^3,4^. IOI can manifest as diffuse or localized mass-like enhancement^5^, while orbital cellulitis often exhibits fat stranding and abscess formation^4,6^. Beyond inflammatory conditions, MRI is invaluable in detecting and characterizing other orbital pathologies, including neoplasms, vascular anomalies, and trauma, making it a cornerstone of comprehensive orbital assessment^7^.

Despite the central role of MRI in diagnosing orbital conditions, interpreting these images presents challenges, particularly due to the absence of a publicly available annotated orbital MRI database, which hampers clinical advancements and the development of fully automated algorithms. In orbital MRI, precise annotation of regions of interest (ROIs) is essential for accurate analysis. For example, in TED, the most common autoimmune orbital disorder, MRI characteristics differ as the disease progresses^8,9^. During the active inflammatory phase, MRI reveals enlarged extraocular muscles with high T2-weighted signal intensity due to edema^3,10^. In contrast, the chronic fibrotic phase shows persistent muscle enlargement with reduced signal intensity due to fibrosis, and sometimes orbital lipid hyperplasia^10,11^.

Accurate ROI annotation captures these pathological and physiological changes, such as increases in muscle volume, which are vital for radiomics quantification. This is particularly valuable in treatment planning, especially when using therapies like Teprotumumab, a monoclonal antibody targeting specific inflammatory pathways^12,13^. By quantifying muscle volume and monitoring changes over time, clinicians can assess therapy efficacy and tailor treatment plans more effectively. T2-weighted imaging (T2WI) is often preferred in TED assessment due to its superior ability to highlight edema, a key indicator of active inflammation, facilitating early diagnosis and timely intervention.

**Fig. 1:**
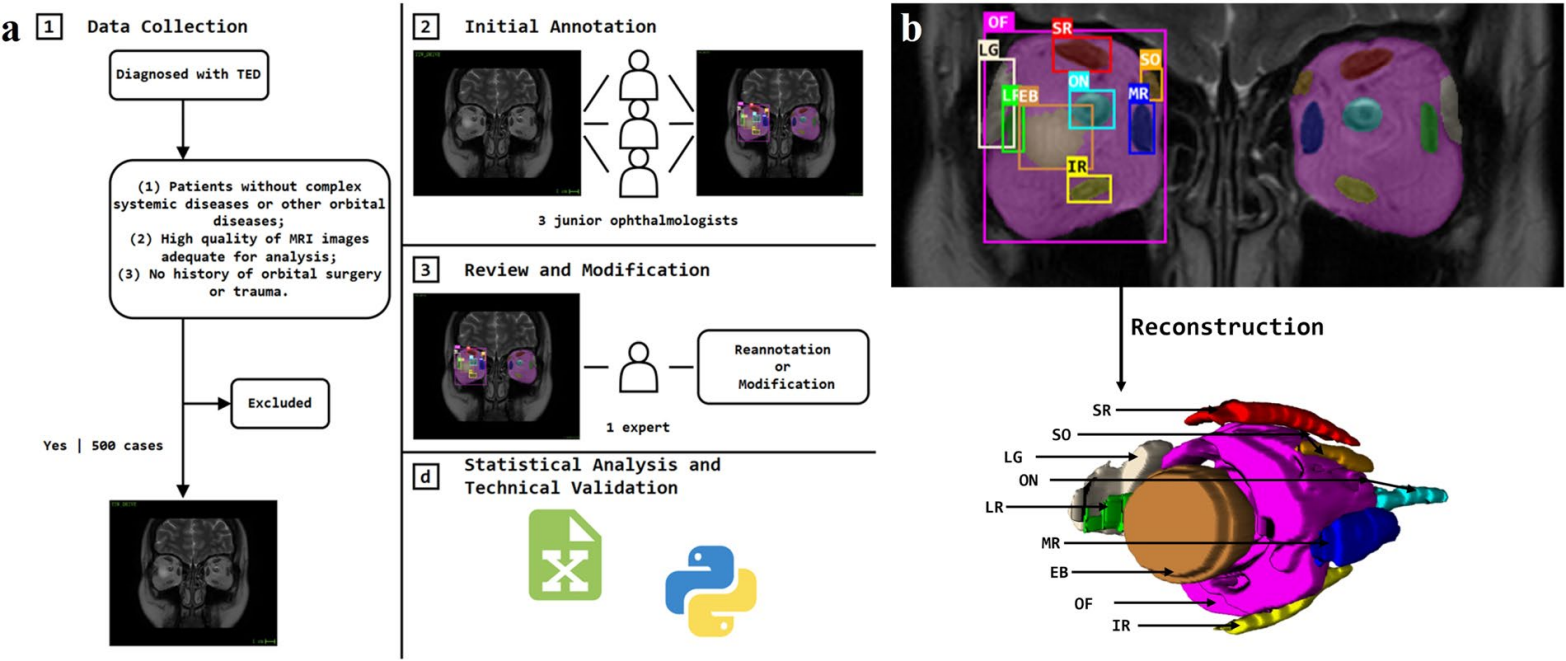
Flowchart of dataset construction and orbital structures **a**. the overall workflow of the dataset construction and statistical analysis; **b**. 2d and 3d illustration of orbital structures. **Abbreviations**: SR = superior rectus (and levator palpebrae superioris complex), SO = superior oblique, LG = lacrimal gland, ON = optic nerve, LR = lateral rectus, MR = medial rectus, EB = eyeball, OF = orbital fat, IR = inferior rectus.

Furthermore, an annotated MRI dataset holds value beyond TED, as it provides a comprehensive reference for various orbital structures, especially soft tissues^3^. This dataset could also be adapted for use in non-TED patients through reinforcement learning techniques, expanding its utility in the field of research on orbital diseases. However, creating such large, annotated datasets for training and validating algorithms is labor-intensive, requiring extensive annotation efforts. Currently, only a few datasets are readily available for open usage, particularly for TED. This is primarily because such datasets can only be gathered and constructed in major clinical centers with large patient populations. Addressing this gap is fundamental for advancing research and enabling performance comparisons of segmentation, classification, and diagnostic algorithms. The panorama of our workflow proposed for dataset construction is illustrated in Fig. 1a below, while Fig. 1b highlights the specific orbital structures included in this process.

In this study, we propose TOM500 (TED Orbital MRI with 500 cases). TOM500 provides a multi-organ annotated orbital T2WI dataset, including 500 cases of TED patients, along with basic clinical characteristics. The dataset provides resources to support future developments in the diagnosis of orbital diseases and the segmentation of orbital structures. Additionally, this dataset establishes a referable paradigm for future datasets of normal orbital data and other orbital diseases.

## Methods

### MRI Data

#### Overall

A total of 500 cases were included in this dataset, with a resolution of 512 × 512 × 20. All images were stored with a bit depth of 12 in NIFTI format.

#### Participants

At their initial visit, clinical and radiological data from 500 anonymous patients with clinically confirmed TED^14^ were collected at Shanghai Ninth People’s Hospital between June 2016 and January 2021. The inclusion criteria were as follows: (1) Patients without complex systemic diseases or other orbital conditions; (2) High-quality MRI images adequate for analysis; (3) No history of orbital surgery or trauma. This manuscript adheres to STROBE guidelines. The study was approved by our Institutional Review Board (SH9H-2021-T246-2), and the requirement for informed consent was waived.

#### MRI data acquisition

Patients were examined using a 3.0 T MRI system (Ingenia, Philips Medical Systems) with a 32-channel head coil. During the scan, patients were positioned in the supine position with their eyes closed. Coronal T2-weighted Turbo Spin-Echo with 90° Flip-Back Pulse (T2-DRIVE) imaging was acquired with the following parameters: repetition time/echo time, 3000 ms/90 ms; image frequency, 127.77 MHz; field of view, 133.33 mm; slice thickness, 3.5 mm; spacing between slices, 3.85 mm; pixel spacing, 0.3125 × 0.3125 mm; acquisition matrix, 320 × 242.

#### Anonymization

All patient-identifiable data have been removed to ensure privacy protection and, therefore, were not included in this dataset.

#### Data preprocessing

Minimal preprocessing with linear normalization was performed using the existing rescale intercept (0028∣1052) and rescale slope (0028∣1053). These parameters define the linear transformation from the stored pixel values to the output pixel values in radiological imaging. This transformation can be expressed by the following equation:

$$U=m\times SV+b$$

where:

*U* is the output value in the desired units,

*m* is the rescale slope,

*SV* is the stored value of the pixel, and

*b* is the rescale intercept.

### Annotation

ROIs were manually segmented on coronal T2WI images using ITK-SNAP software (v. 3.6.0; http://www.itksnap.org). Nine orbital structures, including the optic nerve (ON), orbital fat (OF), lacrimal gland (LG), eyeball (EB), and the separate extraocular muscles (EOMs): superior rectus (SR) (and levator palpebrae superioris complex), inferior rectus (IR), medial rectus (MR), lateral rectus (LR), and superior oblique (SO), were individually contoured using different labels. The contours of each ROI were drawn slice-by-slice, from the emergence of the OF in the anterior orbit to the disappearance of the EOMs in the posterior orbit. For all manual segmentations, three junior orbital radiologists reviewed each MRI and conducted ROI segmentations without knowing the disease status of the participants. Their annotations were fused into one using the Simultaneous Truth and Performance Level Estimation (STAPLE) algorithm and further reviewed by an orbital radiology expert for accuracy. STAPLE performs a pixel-wise combination of multiple input images, each representing a segmentation of the same scene^15–17^. The labelings in these images are weighted relative to each other based on performance estimated by an expectation-maximization algorithm. Through this process, a ground truth segmentation is estimated, and the performance of individual segmentations is evaluated relative to this ground truth. The algorithm iterates until it converges on the quality parameters that maximize the log-likelihood function:

$$\left({p}^{(k)},{q}^{(k)}\right)=arg\mathop{\max }\limits_{p,\,q}E\left[ln\left(f\left(D| \,T,p,q\right)f(T)\right)\left|D,{p}^{(k-1)},{q}^{(k-1)}\right.\right]$$

where:

*p*^(*k*)^, *q*^(*k*)^ are the estimates of the expert performance level parameters at iteration k,

*D* is the matrix describing the binary decisions made for each segmentation at each voxel of the image,

*T* is the hidden binary true segmentation, and

*p*^(*k*−1)^, *q*^(*k*−1)^ are the previous estimates of the expert quality parameters.

The fused annotation was reviewed and, if necessary, modified by a radiology expert.

## Data Records

*TOM500*^18^ is available in the public repository at 10.6084/m9.figshare.27133389. The dataset was released in September 2024, and any future updates will be published with the corresponding release dates. The dataset follows the directory/file structure outlined below:

The ‘image’ folder within the ‘train’ directory contains images from 400 cases, while the ‘image’ folder within the ‘val’ directory contains images from 100 cases. The ‘label’ folders within both the ‘train’ and ‘val’ directories contain ground truth annotations, which were initially reviewed by three junior annotators and subsequently validated by an expert. All files follow a consistent naming convention: ‘[patient id].nii.gz’. The files are in the same format, ensuring better compatibility across devices, making them more suitable for machine learning algorithms and data processing.

## Technical Validation

For analysis, the STAPLE and reviewed ground truth were derived from all annotations. The Dice (D), Jaccard (J), sensitivity (Se), specificity (Sp), precision (Pr), recall (Re), and accuracy (Ac) similarity indices are standard objective measures used to quantify image segmentation performance^19^. These indices are computed based on true positives (TP), false positives (FP), false negatives (FN), and true negatives (TN)^20^. Sensitivity (or true positive rate) is defined as the proportion of true positives correctly identified^21,22^. Specificity (or true negative rate) is defined as the proportion of true negatives correctly identified^21^. Accuracy measures the degree of closeness between the segmentation and the ground truth. The performance indices are computed as follows:

$$\begin{array}{rcl} &  & Sensitivity\,\left(Se\right)=\frac{TP}{TP+FN},\\  &  & Specificity\,\left(Sp\right)=\frac{TN}{TN+FP},\\  &  & Recall\,\left(Re\right)=\frac{TP}{TP+FN},\\  &  & Precision\,\left(Pr\right)=\frac{TP}{TP+FP},\,{\rm{and}}\\  &  & Accuracy\,\left(Ac\right)=\frac{TP+FN}{TP+TN+FP+\,FN}.\end{array}$$

Sensitivity can be misleading, especially in cases of poor segmentation where the segmented area is much larger than the ground truth^23,24^. Therefore, specificity serves as a necessary counterpart to sensitivity. However, in cases of poor segmentation that fail to detect the region of interest (ROI), which may still yield a perfect specificity value, we further analyze the data using the Jaccard and Dice indices. The Jaccard similarity index is the ratio of the intersection to the union of the predicted and true regions. The Dice similarity index is closely related to the Jaccard index, with one being derivable from the other^25^. These indices are defined as follows:

$$\begin{array}{rcl} &  & Jaccard\,index\,\left(J\right)=\frac{| \,A\,\cap \,Gt\,| }{| \,A\,\cup \,Gt\,| },\,{\rm{and}}\\  &  & Dice\,index\,\left(D\right)=\frac{2| \,A\,\cap \,Gt\,| }{\left|\,A\right|+| \,Gt\,| },\end{array}$$

where *A* and *Gt* represent the ROI segmented by the annotator and the ground truth, respectively. By definition, 0 ≤ *J* ≤ 1 and 0 ≤ *D* ≤ 1.

**Fig. 2:**
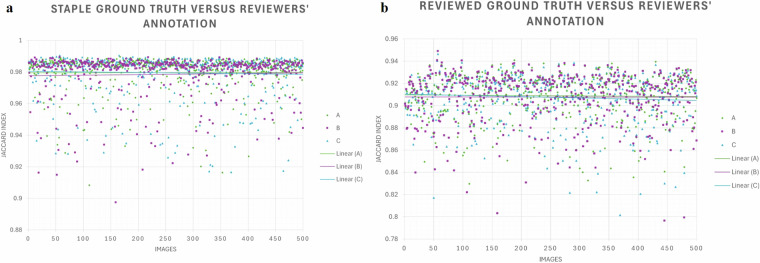
Linear least-squares regression figures of segmentation performance **a**. STAPLE ground truth versus reviewers’ annotation; **b**. Reviewed ground truth versus reviewers’ annotation.

Reviewer-wise segmentation variability analysis, with respect to the STAPLE ground truth and the reviewed ground truth, is presented in Table 1, using the performance indices mentioned above. Linear least-squares regression was employed for visualization (Fig. 2a,b).

**Table 1:** Reviewer-wise segmentation variability analysis.

	Reviewer	Dice	Jaccard	Sens	Spec	Pr	Re	Acc
Versus STAPLE	A	0.990	0.980	0.988	1.000	0.991	0.988	1.000
	B	0.989	0.979	0.991	1.000	0.987	0.991	1.000
	C	0.989	0.979	0.989	1.000	0.990	0.989	1.000
Versus ground truth	A	0.952	0.909	0.951	1.000	0.953	0.951	1.000
	B	0.951	0.908	0.953	1.000	0.950	0.953	1.000
	C	0.951	0.907	0.951	1.000	0.951	0.951	1.000

**Abbreviations**: Sens = sensitivity, Spec = specificity, Pr = precision, Re = recall, Acc = accuracy.

We acquired the average signal intensities of all ten structures involved (including nine orbital structures and the background), along with their volumes. The data were then analyzed using the intraclass correlation coefficient (ICC), with the results presented in Table 2. The ICC is a measure of measurement reliability, with a 95% confidence interval^26^. The closer the ICC is to 1.0, the stronger the linear relationship between two methods for the same measurement^27^. The results show satisfactory outcomes, with all ICCs exceeding 0.95.

**Table 2:** ICC measurements amongst reviewers.

	Label	ICC	F	Df1	Df2	Pval	95%CI
Average signal intensity	BG	1.000	292941.711	499	998	<0.001	[1.000, 1.000]
	SR	1.000	16832.532	499	998	<0.001	[1.000, 1.000]
	LR	0.999	5866.011	499	998	<0.001	[0.999,1.000]
	MR	1.000	20940.504	499	998	<0.001	[1.000, 1.000]
	IR	1.000	17546.598	499	998	<0.001	[1.000, 1.000]
	ON	0.999	3189.803	499	998	<0.001	[0.999,0.999]
	OF	1.000	12224.816	499	998	<0.001	[1.000, 1.000]
	LG	0.999	5102.980	499	998	<0.001	[0.999,0.999]
	SO	1.000	6507.606	499	998	<0.001	[0.999,1.000]
	EB	0.999	3795.076	499	998	<0.001	[0.999,0.999]
Volume	BG	1.000	8208.473	499	998	<0.001	[1.000, 1.000]
	SR	0.998	1464.765	499	998	<0.001	[0.998,0.998]
	LR	0.987	234.772	499	998	<0.001	[0.985,0.989]
	MR	0.999	3672.278	499	998	<0.001	[0.999,0.999]
	IR	0.999	3497.459	499	998	<0.001	[0.999,0.999]
	ON	0.991	325.686	499	998	<0.001	[0.989,0.992]
	OF	0.995	610.397	499	998	<0.001	[0.994,0.996]
	LG	0.984	181.630	499	998	<0.001	[0.981,0.986]
	SO	0.996	679.663	499	998	<0.001	[0.995,0.996]
	EB	0.946	53.075	499	998	<0.001	[0.937,0.954]

**Abbreviations**: ICC = intraclass correlation coefficient, Df = degrees of freedom,95%CI = 95% confidence interval, BG = background.

## Clinical characteristics

Among the 500 enrolled cases, 167 (33.4%) were male and 333 (66.6%) were female, yielding a sex ratio (female to male) of 2:1. The median age was 48 years, consistent with previous epidemiological studies^28–30^. With a median disease duration of 10 months, 77.8% of the cases were non-smokers. Further details can be found in Table 3.

**Table 3:** Clinical characteristics.

Characteristics	Value
Sex	
Male	167 (33.4%)
Female	333 (66.6%)
Age (year)	48 (33, 55)
Disease duration (month)	10 (4, 23)
Smoking	
No	389 (77.8%)
Yes	111 (22.2%)

## Usage Notes

The TOM500 dataset includes clinical data, T2WI scans, and corresponding segmentations. These resources are intended for use by healthcare professionals in ophthalmology and researchers developing computational tools, such as those for patient diagnosis from T2WI images, orbital structure segmentation, or data augmentation. The authors encourage readers to explore a wide range of methods for image enhancement and data analysis using the provided dataset. The dataset is optimized for machine processing, with all analyses conducted in Python using standard libraries such as NumPy, Pandas, and scikit-learn. Maintaining resolution and bit depth is recommended to ensure reliable results. A suggested dataset split is also provided to promote consistency. The code implementing each processing step is presented in Table 4.

**Table 4:** Code implementing each processing step.

Processing step	
1. Anonymization	https://github.com/guest0417/TOM500/blob/main/anonymization.py
2. Alignment	https://github.com/guest0417/TOM500/blob/main/alignment.py
3. Technical validation	https://github.com/guest0417/TOM500/blob/main/technical_validation.py
4. One dimensional feature extraction	https://github.com/guest0417/TOM500/blob/main/one_dimensional_feature_extraction.py

## Data Availability

The code is available under CC0 licensing on GitHub and is accompanied by anonymized patient information in CSV format. For data analysis and validation, Python version 3.9 was used. The dataset was released in September 2024. Any future updates to the database will be published with their respective release dates.

## References

[1] Zhou, M. *et al*. Role of Magnetic Resonance Imaging in the Assessment of Active Thyroid-Associated Ophthalmopathy Patients with Long Disease Duration. *Endocrine Practice* **25**, 1268–1278 (2019). 31412229 10.4158/EP-2019-0133

[2] Yokoyama, N., Nagataki, S., Uetani, M., Ashizawa, K. & Eguchi, K. Role of Magnetic Resonance Imaging in the Assessment of Disease Activity in Thyroid-Associated Ophthalmopathy. *Thyroid* **12**, 223–227 (2002). 11952043 10.1089/105072502753600179

[3] Kahaly, G. J. Imaging in thyroid-associated orbitopathy. *European Journal of Endocrinology* **145**, 107–118 (2001). 11454505 10.1530/eje.0.1450107

[4] Pakdaman, M. N., Sepahdari, A. R. & Elkhamary, S. M. Orbital inflammatory disease: Pictorial review and differential diagnosis. *World J Radiol* **6**, 106–115 (2014). 24778772 10.4329/wjr.v6.i4.106 PMC4000606

[5] Ding, Z. X., Lip, G. & Chong, V. Idiopathic orbital pseudotumour. *Clinical Radiology* **66**, 886–892 (2011). 21546008 10.1016/j.crad.2011.03.018

[6] Diogo, M. C., Jager, M. J. & Ferreira, T. A. CT and MR Imaging in the Diagnosis of Scleritis. *American Journal of Neuroradiology* **37**, 2334–2339 (2016). 27444937 10.3174/ajnr.A4890 PMC7963878

[7] Yuan, Y., Kuai, X.-P., Chen, X.-S. & Tao, X.-F. Assessment of dynamic contrast-enhanced magnetic resonance imaging in the differentiation of malignant from benign orbital masses. *European Journal of Radiology* **82**, 1506–1511 (2013). 23561057 10.1016/j.ejrad.2013.03.001

[8] Lo, C., Ugradar, S. & Rootman, D. Management of graves myopathy: Orbital imaging in thyroid-related orbitopathy. *Journal of American Association for Pediatric Ophthalmology and Strabismus* **22**, 256.e1–256.e9 (2018). 30055270 10.1016/j.jaapos.2018.06.002

[9] Zhang, H. *et al*. Application of Quantitative MRI in Thyroid Eye Disease: Imaging Techniques and Clinical Practices. *Journal of Magnetic Resonance Imaging* **60**, 827–847 (2024). 37974477 10.1002/jmri.29114

[10] Tortora, F. *et al*. Disease Activity in Graves’ Ophthalmopathy: Diagnosis with Orbital MR Imaging and Correlation with Clinical Score. *The Neuroradiology Journal* **26**, 555–564 (2013). 24199816 10.1177/197140091302600509 PMC4202826

[11] Kirsch, E., Hammer, B. & von, A. Graves’orbitopathy: current imaging procedures. *Swiss Medical Weekly* **139**, 618–618 (2009). 19950023 10.4414/smw.2009.12741

[12] Douglas, R. S. *et al*. Teprotumumab for the Treatment of Active Thyroid Eye Disease. *New England Journal of Medicine* **382**, 341–352 (2020). 31971679 10.1056/NEJMoa1910434

[13] Jain, A. P. *et al*. Teprotumumab reduces extraocular muscle and orbital fat volume in thyroid eye disease. *British Journal of Ophthalmology* **106**, 165–171 (2022). 33172865 10.1136/bjophthalmol-2020-317806

[14] Bartalena, L. *et al*. The 2021 European Group on Graves’ orbitopathy (EUGOGO) clinical practice guidelines for the medical management of Graves’ orbitopathy. *European Journal of Endocrinology* **185**, G43–G67 (2021). 34297684 10.1530/EJE-21-0479

[15] Warfield, S., Zou, K. & Wells, W. Validation of image segmentation and expert quality with an expectation-maximization algorithm. in MEDICAL IMAGE COMPUTING AND COMPUTER-ASSISTED INTERVENTION-MICCAI 2002, PT 1 (eds. Dohi, T. & Kikinis, R.) 2488, 298–306 (2002). 10.1007/3-540-45786-0_70 PMC5470604 28626841

[16] Warfield, S. K., Zou, K. H. & Wells, W. M. Simultaneous truth and performance level estimation (STAPLE): an algorithm for the validation of image segmentation. *IEEE Transactions on Medical Imaging* **23**, 903–921 (2004). 15250643 10.1109/TMI.2004.828354 PMC1283110

[17] Rohlfing, T., Russakoff, D. B. & Maurer, C. R. Performance-based classifier combination in atlas-based image segmentation using expectation-maximization parameter estimation. *IEEE Transactions on Medical Imaging* **23**, 983–994 (2004). 15338732 10.1109/TMI.2004.830803

[18] Zhang, H. *et al*. TOM500: A Multi-Organ Annotated Orbital MRI Dataset for Thyroid Eye Disease. *figshare*10.6084/m9.figshare.27133389 (2024). 10.1038/s41597-025-04427-9 PMC11730993 39805915

[19] Wang, Z., Wang, E. & Zhu, Y. Image segmentation evaluation: a survey of methods. *Artificial Intelligence Review* **53**, 5637–5674 (2020).

[20] Chang, H.-H., Zhuang, A. H., Valentino, D. J. & Chu, W.-C. Performance measure characterization for evaluating neuroimage segmentation algorithms. *NeuroImage* **47**, 122–135 (2009). 19345740 10.1016/j.neuroimage.2009.03.068

[21] Taha, A. A. & Hanbury, A. Metrics for evaluating 3D medical image segmentation: analysis, selection, and tool. *BMC Medical Imaging* **15**, 29 (2015). 26263899 10.1186/s12880-015-0068-x PMC4533825

[22] Lukac, P. *et al*. Simple comparison of image segmentation algorithms based on evaluation criterion. in Proceedings of 21st International Conference Radioelektronika 2011 1–4 (2011).

[23] Dey, N., Rajinikanth, V., Ashour, A. S. & Tavares, J. M. R. S. Social Group Optimization Supported Segmentation and Evaluation of Skin Melanoma Images. Symmetry 10, (2018).

[24] Chouhan, S. S., Kaul, A. & Singh, U. P. Soft computing approaches for image segmentation: a survey. *Multimedia Tools and Applications* **77**, 28483–28537 (2018).

[25] Garcia-Lamont, F., Cervantes, J., López, A. & Rodriguez, L. Segmentation of images by color features: A survey. *Neurocomputing* **292**, 1–27 (2018).

[26] Shrout, P. E. & Fleiss, J. L. Intraclass correlations: Uses in assessing rater reliability. *Psychological Bulletin* **86**, 420–428 (1979). 18839484 10.1037//0033-2909.86.2.420

[27] Rousson, V. Assessing inter-rater reliability when the raters are fixed: Two concepts and two estimates. *Biometrical Journal* **53**, 477–490 (2011). 21425184 10.1002/bimj.201000066

[28] Jaru-Ampornpan, P., Cheng, Y., Jiao, Q. & Douglas, R. S. Thyroid eye disease in Asians and recent advances in management. *Chinese journal of endocrinology and metabolism* **36**, 541–562 (2020).

[29] Tsai, C.-C., Kau, H.-C., Kao, S.-C. & Hsu, W.-M. Exophthalmos of patients with Graves’ disease in Chinese of Taiwan. *Eye* **20**, 569–573 (2006). 15905866 10.1038/sj.eye.6701925

[30] Lim, S. L. *et al*. Prevalence, Risk Factors, and Clinical Features of Thyroid-Associated Ophthalmopathy in Multiethnic Malaysian Patients with Graves’ Disease. *Thyroid* **18**, 1297–1301 (2008). 19012471 10.1089/thy.2008.0044

